# Establishment of a graphene quantum dot (GQD) based steroid binding assay for the nuclear progesterone receptor (pgr)

**DOI:** 10.1016/j.bbrep.2024.101691

**Published:** 2024-03-25

**Authors:** Md. Forhad Hossain, Shakhawat Hossain, Md. Maisum Sarwar Jyoti, Yuki Omori, Saokat Ahamed, Toshinobu Tokumoto

**Affiliations:** a Department of Bioscience, Graduate School of Science and Technology, National University Corporation, Shizuoka University, 836 Ohya, Suruga-ku, Shizuoka, 422-8529, Japan; b Biological Science Course, Department of Science, Graduate School of Integrated Science and Technology, Shizuoka University, 836 Ohya, Suruga-ku, Shizuoka, 422-8529, Japan

**Keywords:** Graphene quantum dots, Nuclear progesterone receptor, Progesterone, FRET, Steroids

## Abstract

Previously, we established a homogeneous assay for membrane progesterone receptor alpha (mPRα) ligands by conjugating semiconductor nanoparticles known as graphene quantum dots (GQDs) to mPRα. When mixed with a progesterone-BSA-fluorescein isothiocyanate conjugate (P4-BSA-FITC), fluorescence occurred by fluorescence resonance energy transfer (FRET) but was reduced by the ligand-receptor binding activity. The established way showed ligand specificity as mPRα protein. In this study, we tried to establish the same way for nuclear progesterone receptor (Pgr). The ligand-binding domain (LBD) of zebrafish Pgr (zPgrLBD) was expressed as a fusion protein with glutathione S-transferase (GST) (GST-zPgrLBD). The recombinant protein was then purified and coupled with GQDs to produce GQD-conjugated GST-zPgrLBD (GQD-GST-zPgrLBD). When mixed with a P4-BSA-FITC and activated by 370 nm light, fluorescence at 520 nm appeared by FRET mechanism. Fluorescence at 520 nm was reduced by adding free progesterone to the reaction mixture. Reduction of fluorescence was induced by zPgr ligands but not by steroids or chemicals that do not interact with zPgr. The results showed the formation of a complex of GQD-GST-zPgrLBD and P4-BSA-FITC with ligand-receptor binding. The binding of the compounds was further confirmed by a radiolabeled steroid binding assay. A homogenous ligand-binding assay for nuclear progesterone receptor has been established.

## Highlights

• The ligand-binding domain (LBD) of the zebrafish progesterone receptor (zPgr) was produced as a recombinant protein.
• ZPgrLBD was conjugated with graphene quantum dots (GQDs).
• A high-throughput screening assay (HTS) for zPgr based on FRET with FITC has been established.
• The specificity of the HTS assay was confirmed using zPgr ligands.

## 1 Introduction

Progesterone (P4) is a steroid hormone produced by the placenta, ovaries, and adrenal glands that is essential for maintaining pregnancy and controlling the menstrual cycle [1,2]. Abortion and premature birth are caused by a problem with progesterone biosynthesis and secretion [3,4]. P4 also has important physiological roles in the control of several tissues outside the reproductive system, including bone, the cardiovascular system, the brain, and the mammary gland [[5], [6], [7], [8]]. Synthetic steroidal progestins are widely used as therapeutic agents in the treatment of infertility, combined hormone replacement therapy and many other endocrine disorders [9,10]. Progestins for hormone therapy in Alzheimer's disease have recently attracted attention [11]. Progestins regulate physiological processes through the nuclear progesterone receptor (Pgr), progesterone receptor membrane components (Pgrmcs), and membrane progesterone receptors (mPRs) [[12], [13], [14], [15], [16]].

The mechanisms of ovulation induction by progestins have been well studied in fish models [17]. Ovulation is induced by an increase in luteinizing hormone (LH), which induces the production of the progestin 17 alpha, 20 beta-dihydroxy-4-pregnen-3-one (17,20β-DHP) in follicle cells and makes the eggs ready for fertilization before spawning [18]. As described above, finding a wide range of physiological functions of progestins in a variety of tissues, development of therapeutic agents is still necessary. Also, finding of other receptors for progestins it raises the necessity of analysis of ligand binding specificity assay on different receptors [19]. In fact, recently we have purified natural compounds that act on mPRα from seaweed [20]. It is necessary to analyze the reactivity of these compounds with Pgr.

The receptor for P4, Pgr, was identified and its protein structure was resolved into five domains [[21], [22], [23]]. Zebrafish Pgr contained 617 amino acid residues identified from ovary and testis [24,25]. It showed strong homology to other vertebrate Pgr and had five nuclear steroid receptor-specific domains. The ligand- and DNA-binding domain of zebrafish Pgr showed the highest sequence similarity to eel Pgr and 67%–83% amino acid sequence identity of the ligand-binding domain (LBD) of zebrafish Pgr with known Pgr of other vertebrates [24]. In contrast to the Japanese eel (*Anguilla japonica*) and *Xenopus laevis*, which produce two Pgr proteins from separate loci that differ significantly in their amino acid sequences, the zebrafish showed a single locus for Pgr [[26], [27], [28], [29]].

In 1997, Klotz et al. [30] introduced the first yeast and mammalian cell-based assay for Pgr transactivation, expressing the human Pgr. This seminal study focused on investigating the effect of DDT on Pgr transactivation.

An increasing number of both natural compounds and anthropogenic chemicals, particularly environmental pollutants, exhibit receptor-binding properties and are referred to as endocrine disrupting chemicals (EDCs) [31,32]. Most EDCs are designed to act as receptor agonists or antagonists and interfere with the body's normal physiological system [33,34].

The Pgr Chemical Activated LUciferase gene eXpression (CALUX) reporter gene bioassay has been used to detect anti-progestagenic activities of brominated flame retardants, polycyclic musks, and UV filters [35,36]. It has also been used to identify progestagenic activities in wastewater-derived water [37] and (anti)progestagenic activities in surface and wastewater [38]. The assay has also been used to determine the progestagenic activities of anabolic androgenic steroids used in sports doping [39], a wide variety of compounds [40], and as a pseudo-immunoassay for the detection of progestins [41,42]. Recently, several advanced analytical techniques have been developed for the detection of EDCs [[43], [44], [45]]. These are mainly monitored by *in vitro* reporter gene bioassays based on human Pgr. However, results obtained from some human *in vitro* bioassays may not be relevant to aquatic animals, especially fish, and it has been reported that human Pgr *in vitro* bioassays could not predict fish Pgr activities in the environment [46].

The amino acid similarity between the LBD of Pgr in certain fish species and human Pgr is rather low, approximately 65–69% [47,48]. Consequently, the majority of synthetic progestins that are agonists of human Pgr act as antagonists of fish Pgr [49,50]. According to Garoche et al. [50], the human agonist ligands progestin and progesterone were found to stimulate luciferase activity in both cell lines in a concentration-dependent manner. However, the natural zebrafish progestin 17,20β-DHP activated zebrafish Pgr but not human Pgr. Therefore, data obtained from *in vitro* bioassays using human Pgr (such as (anti-)PR-CALUX) to determine (anti-)progestogenic activity may not be applicable to fish [46]. To identify the fish Pgr-interacting EDCs, it is necessary to develop a fish specific assay.

Fish Pgr based on *in vitro* reporter gene bioassays have been developed [[48], [49], [50]]. Although these assays demonstrate high sensitivity, their limitations stem from their substantial cost and time-consuming nature.

Previously, we have established a fluorescence-based GQD-hmPRα [51] high-throughput assay system by coupling human membrane progesterone receptor α (hmPRα) with graphene quantum dots (GQDs). As donor fluorophores in Förster resonance energy transfer (FRET), quantum dots are used in biology. Due to their high extinction coefficient and spectrum purity, these fluorophores are superior to molecular fluorophores in this application [52]. The established GQD-hmPRα assay method allows us to identify molecules that interact with the hmPRα, which performs its functions in a non-genomic manner. Recently, we developed a homogeneous assay system for goldfish membrane progesterone receptor α (GmPRα) conjugated with GQDs to screen for compounds that affect oocyte maturation [53]. Functional expression of the LBD of human Pgr as a fusion protein has been reported [34,54,55]. Therefore, we attempted to express zebrafish PgrLBD as a fusion protein and establish a GQD assay system. The established GQD-zPgr assay system allows both agonist and antagonist screening on Pgr.

## 2 Materials and Methods

### 2.1 Materials

Citric acid, *N*-(3-Dimethylaminopropyl)-*N*′-ethylcarbodiimide hydrochloride, *N*-hydroxysuccinimide and steroids (cortisol, progesterone, testosterone, 17β-estradiol) were purchased from Sigma Aldrich Chemicals (St. Louis, MO). 17,20β-DHP was purchased from Toronto Research Chemicals (Toronto, Canada). Org OD 02 was obtained from AXON Medchem BV (Groningen, Netherlands). To purchase more chemicals, Wako Pure Chemical Industries, Ltd. (Osaka, Japan) was contacted. From Spectra Laboratories Inc. in the USA, we purchased Spectra 3.5 kDa dialysis membranes.

### 2.2 Preparation of GST-zPgrLBD

**Fig. 1:**
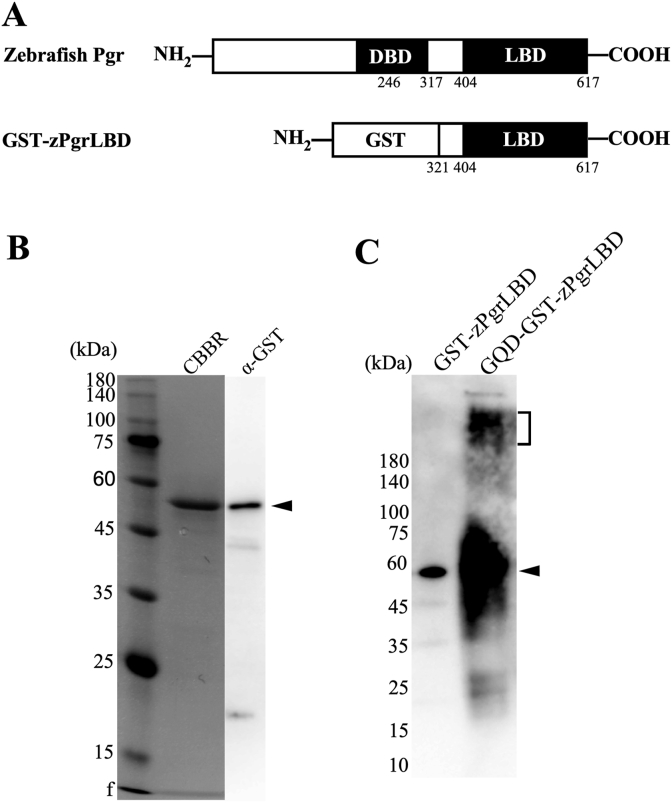
Expression and purification of the ligand-binding domain (LBD) of zebrafish Pgr as a fusion protein with GST and production of GQD-coupled GST-zPgrLBD. A. A diagram of the produced recombinant GST-fusion zPgrLBD. The predicted ligand-binding domain at the *C*-terminus is indicated by black boxes. The GST tag at the *N*-terminus is indicated by a white box. B. SDS-PAGE analysis of purified recombinant GST-zPgrLBD. Protein bands were detected by CBBR staining (CBBR) or immunostained with anti-GST-tag antibody (α-GST). C. Western blot analysis of GST-zPgrLBD and GQD-GST-zPgrLBD. The protein band of GST-zPgrLBD is indicated by an arrowhead. The bands of GQD-GST-zPgrLBD are indicated by a parenthesis.

A portion of the zebrafish Pgr ligand-binding domain (zPgrLBD) PCR product was amplified by PCR using restriction enzyme site containing primers. The amplified cDNA was digested with *Bam*HI and *Xho*I and inserted into the GST vector (Fig. 1A). The construct was transformed into *E. coli* (pBluescript). Expression of GST-zPgrLBD was induced by IPTG (NZCYM medium) at 37 °C for 4 h. Cells were harvested by centrifugation and resuspended in ice-cold lysis buffer (50 mM sodium phosphate, 1 mM PMSF, 1 mM EDTA, 5% glycerol, pH 7.4). Cells were then disrupted by sonication. The soluble and insoluble fractions were then separated by centrifugation at 13,000×*g* for 10 min at 4 °C. GST-zPgrLBD was purified from the insoluble fraction by SDS-PAGE as previously described [56,57]. Purified GST-zPgrLBD proteins were stored at −30 °C. All purification steps were performed at 4 °C.

### 2.3 Preparation of GQDs

The GQDs were prepared by direct pyrolysis of citric acid as described [58].

### 2.4 Preparation of GQDs coupled GST-zPgrLBD

The GQD-labeled GST-zPgrLBD (GQD-GST-zPgrLBD) was prepared by coupling the amine group/N-terminus of GST-zPgrLBD with the carboxylic acid group of GQDs using the standard *N*-ethyl-*N*'-(3-(dimethylamino) propyl) carbodiimide (EDC)/N-hydroxysuccinimide (NHS) reaction as described previously [51]. The prepared GQD-GST-zPgrLBD was stored at −30 °C.

### 2.5 Preparation of FITC-labeled progesterone-coupled BSA (P4-BSA-FITC)

P4-BSA-FITC was prepared using the previously established standard reaction for progesterone-linked BSA (4-pregnene-3,20-dione 3-*O*-carboxymethyloxime/BSA, Steraloids Inc. USA) [51].

### 2.6 Binding assay using GQD-GST-zPgrLBD and P4-BSA-FITC

The binding assay using GQD-GST-zPgrLBD and P4-BSA-FITC was conducted as previously described [51]. The final concentrations of GQD-GST-zPgrLBD at 9 μg/ml, P4-BSA-FITC at 14 μg/ml BSA was added and mixed.

### 2.7 Radiolabeled ligand binding assays

The physical binding of GQD-GST-zPgrLBD with compounds was further confirmed by an established steroid binding assay using ^3^H-labeled steroids [59].

## 3 Results

### 3.1 Preparation of GQD-GST-zPgrLBD

Recombinant GST-zPgrLBD was purified as a single band with an expected size of 57 kDa (Fig. 1B). GST fusion was confirmed by Western blot using anti-GST monoclonal antibody (Fig. 1B). GQDs were prepared manually from citric acid. GQD-GST-zPgrLBD was prepared by EDC/NHS coupling as described in Materials and Methods. The preparation of GQD-GST-zPgrLBD was confirmed by Western blot (Fig. 1C).

The spectrometric characteristics of the GQD-GST-zPgrLBD prepared in this study were the same as those of the previously reported GQD-hmPRα. The maximum fluorescence intensity of the synthesized GQD-GST-zPgrLBD was observed at an excitation wavelength of 370 nm, while the peak fluorescence of the compound was observed at 470 nm (data not shown).

### 3.2 Specific binding of progesterone in P4-BSA-FITC with GQD-GST-zPgrLBD

**Fig. 2:**
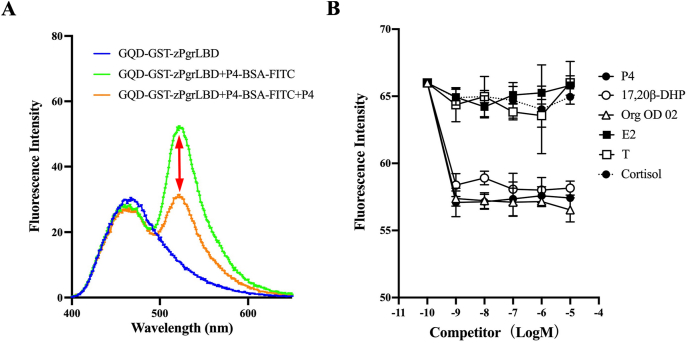
Fluorescence characteristics of GQD-GST-zPgrLBD and competition of binding of P4-BSA-FITC with GQD-GST-zPgrLBD by steroids and their analogues. (A) The fluorescent scanning pattern of free GQD-GST-zPgrLBD is indicated in blue line. Fluorescent scanning pattern of the reaction mixture with (orange line) or without free P4 (green line). The difference of fluorescence at 520 nm caused by the addition of free P4 is indicated by the double-sided arrow. (B) The dose-dependent effects of steroids (progesterone (P4), 17β-estradiol (E2), testosterone (T), cortisol) and their analogues (17,20β-DHP and Org OD 02) were determined using the established assay. An assay was performed in triplicate for each compound. (For interpretation of the references to colour in this figure legend, the reader is referred to the Web version of this article.)

**Fig. 3:**
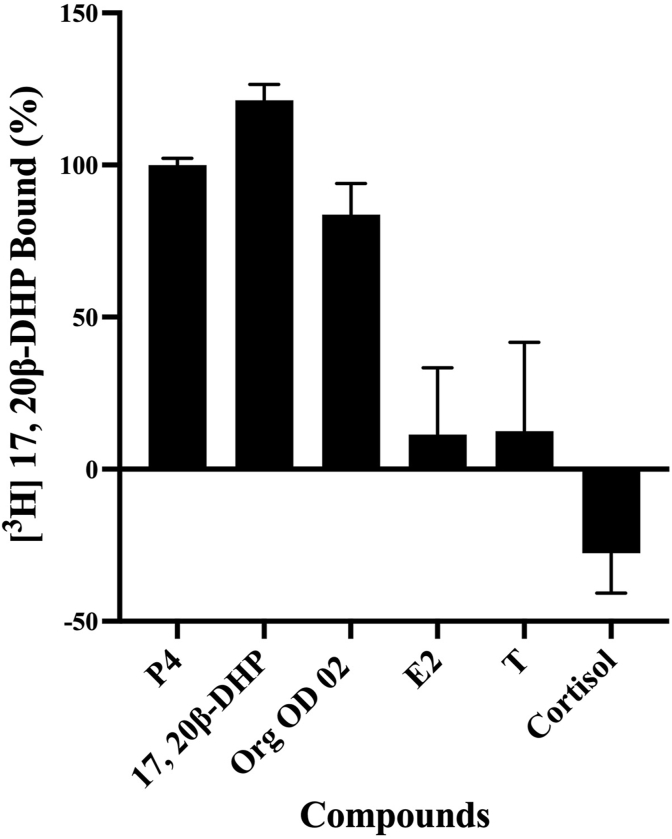
Competition by steroids and progestins for binding to GQD-GST-zPgrLBD. Samples were incubated with 2 nM [^3^H]-17,20β-DHP and 1 μM of competitor. An assay was performed in triplicate for each competitor. The average of the three assays is indicated with standard deviation. Competition for [^3^H]-17,20β-DHP binding are expressed as a percentage of maximum specific progesterone (P4) binding. The competitors analyzed were progesterone (P4); 17,20β-DHP; Org OD 02; 17β-estradiol (E2); testosterone (T); Cortisol.

We then tried to detect the binding of GQD-GST-zPgrLBD and P4-BSA-FITC. When we set the excitation wavelength for the reaction mixture to 370 nm, which was the maximal excitation wavelength of GQD-GST-zPgrLBD, fluorescence intensity at 520 nm was recorded. This was lowered by adding free progesterone to the reaction mixture (Fig. 2A). Thus, we concluded that the fluorescence of GQD-GST-zPgrLBD activated the binding of FITC on BSA by a FRET mechanism. And this fluorescence intensity was reduced by dissociation of P4-BSA-FITC by competitive binding of free P4 to GQD-GST-zPgrLBD (Fig. 2A). Assays for various steroids and progesterone-related compounds have further confirmed the specificity of this assay (Fig. 2B). Progesterone, a known Pgr ligand, and 17,20β-DHP all demonstrated competitive binding activity against P4-BSA-FITC. Recombinant GST-zPgrLBD also showed binding activity for the natural ligand 17,20β-DHP. In addition, a P4 analog known as mPRα selective agonist, Org OD 02, showed binding activity. 17β-estradiol, testosterone, and cortisol, in contrast, did not have any activity even at high concentrations. Binding of GQD-GST-zPgrLBD to these compounds was further confirmed by binding assay with ^3^H-labeled steroid, ^3^H-17,20β-DHP (Fig. 3). All compounds (P4, 17,20β-DHP and Org OD 02) with fluorescence-reducing activity in the GQD-FITC assay showed specific binding activity in the radiolabeled binding assay. In contrast, the compounds without fluorescence-reducing activity (E2, T and cortisol) showed no binding activity. Thus, we concluded that we were able to detect the binding of Pgr and its ligands.

## 4 Discussion

Previously, we were able to purify a relatively large amount of human mPRα protein with hormone-binding activity [60]. We developed a method to identify the precise steroid binding to the mPRα protein using purified recombinant proteins, which is anticipated to facilitate high-throughput screening of mPRα agonists or antagonists [51]. In this study, we succeeded in establishing the same method for zPgr by producing GQD coupled to the ligand-binding domain of zPgr.

The selectivity of progestin-specific steroids has been confirmed. Of the four steroids, only progesterone and its analogues showed binding. 17β-estradiol, testosterone and cortisol showed no binding activity, according to the results of a binding assay using ^3^H-labeled steroids and recombinant proteins expressed in cancer cells [61,62]. The types of substance groups acting on Pgr have also been reported by transcription induction studies developed to investigate the effects of steroids and EDCs on Pgr [50,63]. The present results are also consistent with these studies. The physical binding of GQD-GST-zPgrLBD with compounds was further confirmed by an established steroid binding assay using ^3^H-labeled steroids [59]. These findings imply that our established binding assay can identify the precise chemicals that bind to the nuclear progesterone receptor binding site.

The progestin known as mPRα selective agonist, Org OD 02, showed binding activity on zPgr. Org OD 02 is known as an mPR selective agonist have a higher potency than P4 in the G protein activation assay that target mPRα dependent response [61], but it showed high binding affinity for the GST-zPgrLBD in this study. Also in our previous *in vivo* oocyte maturation and ovulation induction experiments in zebrafish, Org OD 02 induced ovulation [64]. It has been shown in *pgr* gene knockout zebrafish that ovulation induction in zebrafish is a genomic response mediated by Pgr. These results suggest that Org OD 02 acts on Pgr to induce ovulation. The GQD assay in the present study confirmed that Org OD 02 binds to Pgr, demonstrating its ovulation-inducing effect.

Oocyte maturation is known to be induced by mPR-mediated nongenomic actions by progestins [[65], [66], [67]]. To clarify the specificity of agents, act on Pgr and mPR, we also established a GQD-GmPRα assay using the goldfish mPRα to screen the fish spawning inducers [53].

Our GQD-zPgr assay system allows both agonist and antagonist screening, and selected Pgr-interacting compounds can then be further evaluated using the zebrafish *in vivo* and *in vitro* assay system to determine their effects on fish oocyte maturation and ovulation [68]. Our established GQD-zPgr homogenous assay system will be highly feasible for screening EDCs associated with ovulation.

In this study, a binding assay for zebrafish Pgr was developed, allowing high-throughput screening (HTS) of compounds with Pgr-acting properties. This homogenous assay will lead to the identification of new chemicals for pharmaceuticals.

## CRediT authorship contribution statement

**Md. Forhad Hossain:** Writing – original draft, Validation, Methodology, Formal analysis, Data curation. **Shakhawat Hossain:** Methodology, Formal analysis, Data curation. **Md. Maisum Sarwar Jyoti:** Methodology, Data curation, Conceptualization. **Yuki Omori:** Formal analysis, Data curation. **Saokat Ahamed:** Formal analysis, Data curation. **Toshinobu Tokumoto:** Writing – review & editing, Supervision, Software, Resources, Project administration, Funding acquisition, Data curation, Conceptualization.

## Declaration of competing interest

The authors declare that there no conflicts of interest.

## Data Availability

Data will be made available on request.
